# Low-grade appendiceal mucinous neoplasm in an uncomplicated Amyand’s hernia: a case report

**DOI:** 10.1093/jscr/rjag796

**Published:** 2026-09-21

**Authors:** Pietro Calabrese, Francesco Cobellis, Maria Spagnuolo, Luigi Cobellis

**Affiliations:** Surgical Unit Cobellis Clinical Hospital, Vallo della Lucania, Italy; Department of General Surgery, Transplantation and Gastroenterology, Federico II University Hospital, Naples, Italy; Surgical Unit Cobellis Clinical Hospital, Vallo della Lucania, Italy; University of Padua, Padua, Italy; Department of General Surgery, Transplantation and Gastroenterology, Federico II University Hospital, Naples, Italy; Surgical Unit Cobellis Clinical Hospital, Vallo della Lucania, Italy

**Keywords:** Amyand’s hernia, low-grade appendiceal mucinous neoplasm, appendiceal neoplasm, inguinal hernia, case report

## Abstract

Amyand’s hernia, defined as the presence of the vermiform appendix within an inguinal hernia sac, is an uncommon surgical finding. The association with appendiceal neoplasms is exceedingly rare, particularly in the absence of inflammatory signs. We report the case of a 71-year-old male with a longstanding right inguinoscrotal hernia presenting with progressive discomfort. An open hernia repair revealed the cecum and a macroscopically non-inflamed appendix within the hernia sac. Appendectomy was performed, followed by sutureless mesh hernioplasty. Histopathological examination unexpectedly demonstrated a low-grade appendiceal mucinous neoplasm with free resection margins. The postoperative course was uneventful, the patient remained asymptomatic at follow-up. This case suggests that even a clinically silent appendix within an Amyand’s hernia may harbor occult neoplastic pathology. Appendectomy should therefore be considered in adult patients to avoid missed diagnoses with potential oncological implications.

## Introduction

Amyand’s hernia is a rare condition characterized by the presence of the vermiform appendix within an inguinal hernia sac, accounting for ~0.2%–1% of all inguinal hernias [1, 2]. Although first described by Claudius Amyand in 1735, it remains an infrequent intraoperative finding, most often diagnosed incidentally during hernia repair. Acute appendicitis occurring within an Amyand’s hernia is even less common and represents a small fraction of all cases of appendicitis.

Low-grade appendiceal mucinous neoplasms (LAMN) are rare epithelial tumours of the appendix, typically discovered incidentally following appendectomy performed for unrelated indications [3]. These lesions are characterized by low-grade cytological atypia and a generally indolent biological behaviour; however, progression to pseudomyxoma peritonei may occur in cases of perforation or incomplete resection. As a result, accurate diagnosis and complete excision are crucial.

The coexistence of Amyand’s hernia and appendiceal neoplasia is exceptionally rare. The majority of published reports describe appendiceal tumours associated with inflammation, incarceration, or perforation, often discovered during emergency surgery [4–7]. The presence of a clinically silent, macroscopically normal appendix harboring a LAMN within an uncomplicated Amyand’s hernia is particularly uncommon. We present such a case to emphasize the importance of intraoperative decision-making and routine histological assessment.

## Case report

A 71-year-old male presented with a progressively enlarging right inguinoscrotal swelling associated with discomfort. The mass had been present for ~2 years, with worsening symptoms over the previous four months. His medical history included an acute myocardial infarction with ST-segment elevation in 2020 treated with three coronary angioplasties, recurrent atrial fibrillation requiring multiple cardioversions, and chronic anticoagulant therapy.

Physical examination revealed a large, reducible right inguinoscrotal hernia without local inflammatory signs. Abdominal examination was otherwise unremarkable, and McBurney’s sign was negative. Preoperative imaging was not performed, as the diagnosis of inguinal hernia was made clinically.

**Figure 1:**
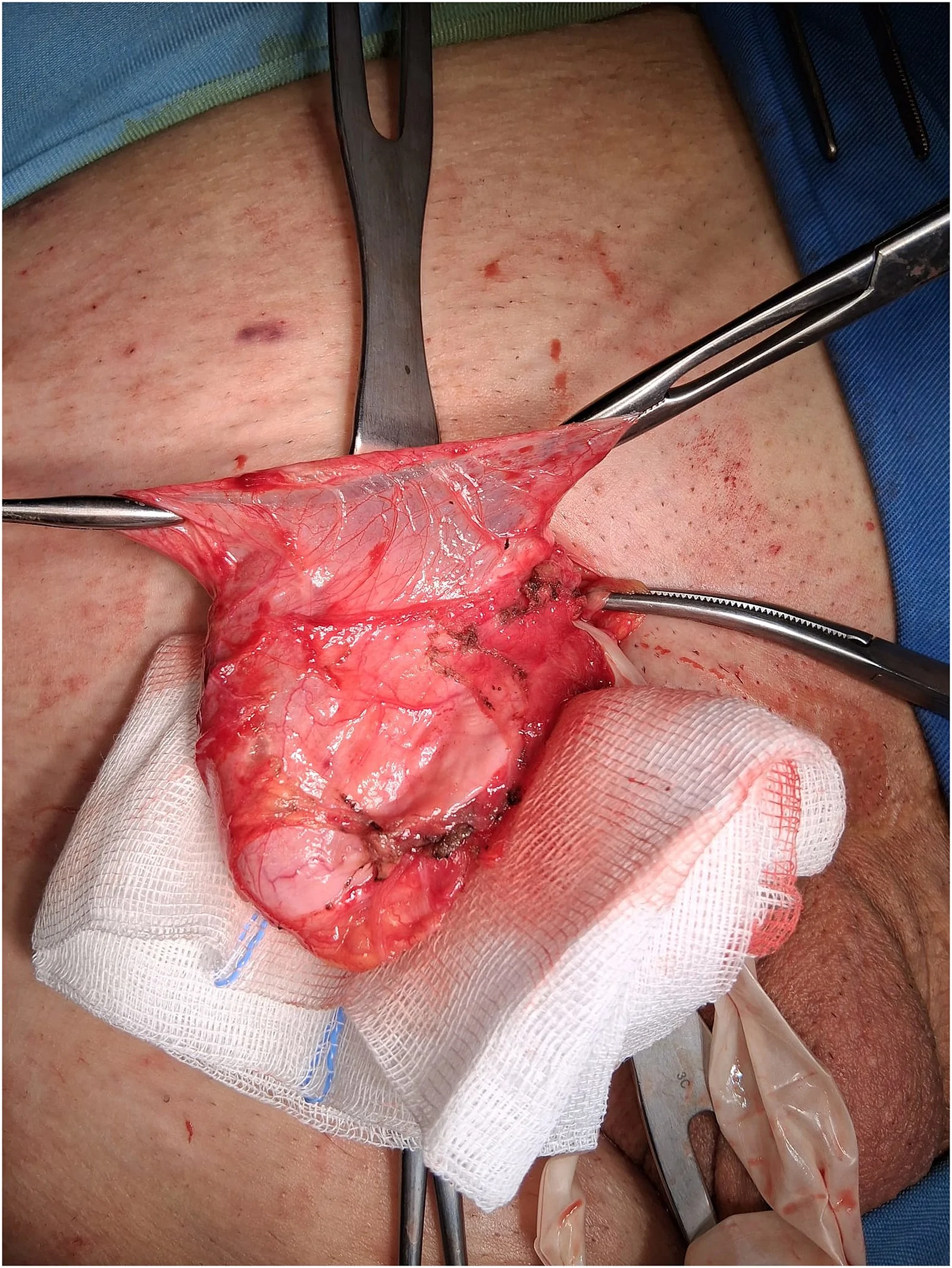
Intraoperative view of the hernia sac containing the vermiform appendix.

**Figure 2:**
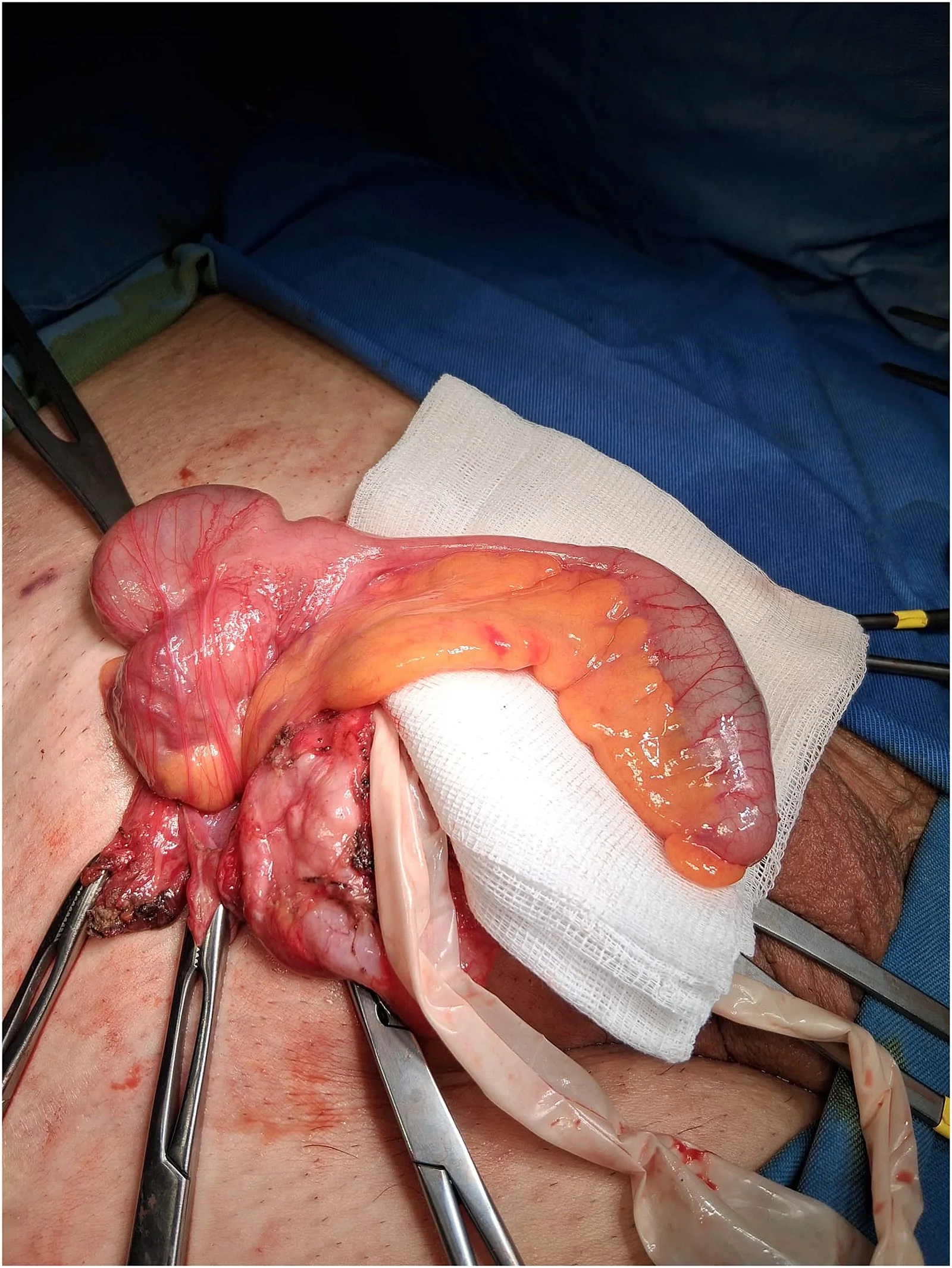
Intraoperative view after exteriorization of the hernia contents. No signs of inflammation are evident.

An open surgical approach was undertaken. Intraoperatively, the hernia sac contained part of the cecum and the entire appendix (Fig. 1). The appendix measured ~15 cm in length and appeared macroscopically non-inflamed (Fig. 2). A firm intraluminal structure was noted and initially presumed to be a coprolite.

Appendectomy was performed using a tobacco-pouch closure of the appendiceal stump. Given the absence of contamination or inflammation, the procedure was completed with a sutureless mesh hernioplasty according to the Trabucco technique.

Histopathological examination revealed a LAMN with free resection margins. The postoperative course was uneventful. At 6-month follow-up, the patient remained asymptomatic, with no evidence of hernia recurrence or disease-related complications.

The patient’s symptoms began ~2 years prior to presentation, with progressive worsening over the four months preceding surgery. Surgical repair was performed on 28 November 2025. Histopathological diagnosis of LAMN was established ~1 week postoperatively. The patient was followed up at 3 and 6 months postoperatively, with no evidence of recurrence or complications at either visit.

## Discussion

Amyand’s hernia represents a rare surgical entity, and the presence of appendiceal neoplasia within the hernia sac is exceedingly uncommon. Most reported cases of appendiceal neoplasia within Amyand’s hernia describe malignant tumours—including adenocarcinoma and carcinoid tumours—discovered in the context of acute inflammation, incarceration, or perforation [4–7]. Montali *et al.* similarly emphasized the oncologic risk that may be concealed within the hernia sac, irrespective of histological subtype [8]. In contrast, reports specifically describing LAMN within an uncomplicated Amyand’s hernia remain exceedingly rare [9, 10]. Our patient, however, presented with a chronic, uncomplicated inguinoscrotal hernia and no clinical or intraoperative signs suggestive of appendiceal pathology.

Primary appendiceal tumours are rare, and LAMN represents a small subset characterized by low-grade histological features and favourable prognosis following complete resection [3]. Previously reported cases of LAMN within an Amyand’s hernia often demonstrated macroscopic abnormalities such as mucoceles or inflammatory changes, or were identified incidentally during surgery for other abdominal conditions [9, 10]. In our case, the appendix appeared entirely normal on gross inspection, and the diagnosis was established exclusively through histopathological analysis.

This finding has important implications for surgical management. Losanoff and Basson proposed a classification system for Amyand’s hernia, suggesting that appendectomy may be optional in Type 1 cases with a normal appendix [2]. However, our experience indicates that this approach may underestimate the risk of occult pathology in adult patients. Our report supports consideration of appendectomy when the appendix is encountered within an inguinal hernia sac, even in the absence of inflammation, to avoid missed diagnoses with potential oncological consequences.

The use of mesh in Amyand’s hernia remains controversial due to concerns about infection. In our case, the absence of appendiceal inflammation or contamination allowed for safe mesh placement, with no postoperative complications observed. This is consistent with selected reports supporting mesh hernioplasty in non-contaminated cases.

For surgeons, particularly those early in their career, this case underscores the importance of maintaining a high index of suspicion and performing appendectomy when appropriate, even in seemingly benign presentations.

The principal strength of this report lies in the histopathological confirmation of LAMN in a macroscopically normal appendix within an uncomplicated Amyand’s hernia, a combination only rarely documented in the literature. This finding directly informs intraoperative decision-making regarding appendectomy in similar presentations. The main limitation is the single-case design, which precludes generalizable conclusions regarding the true incidence of occult appendiceal neoplasia in Amyand’s hernia; preoperative imaging was also not performed, as the diagnosis was made clinically, and routine cross-sectional imaging might have altered the surgical approach in selected cases. Larger case series or systematic reviews are needed to better define the actual risk and to guide evidence-based recommendations on appendectomy in Type 1 Amyand’s hernia.

The coexistence of a clinically silent Amyand’s hernia and a low-grade appendiceal mucinous neoplasm is exceedingly rare. This case demonstrates that a macroscopically normal appendix may conceal occult neoplastic disease. In adult patients, appendectomy should be considered when the appendix is encountered within an Amyand’s hernia to ensure accurate diagnosis and appropriate histological assessment.
